# Role of non-statin lipid-lowering therapy in coronary atherosclerosis regression: a meta-analysis and meta-regression

**DOI:** 10.1186/s12944-020-01297-5

**Published:** 2020-05-27

**Authors:** Walter Masson, Martin Lobo, Daniel Siniawski, Graciela Molinero, Gerardo Masson, Melina Huerín, Juan Patricio Nogueira

**Affiliations:** 1 Council of Epidemiology and Cardiovascular Prevention, Argentine Society of Cardiology, Azcuenaga 980, C1115AAD Buenos Aires, Argentina; 2 Argentine Society of Lipids, Ambrosio Olmos 820, X5000JGQ Córdoba, Argentina; 3 Av. Dr. Luis Gutniski 3200, 3600 Formosa, Argentina

**Keywords:** Intravascular ultrasound, Coronary atherosclerosis plaque, Ezetimibe, PCSK9 inhibitors, Non-statin therapy, Atherosclerosis regression, Meta-analysis

## Abstract

**Background:**

Several studies have investigated the association between non-statin lipid-lowering therapy and regression of atherosclerosis. However, these studies were mostly small and their results were not always robust. The objectives were: (1) to define if a dual lipid-lowering therapy (statin + non-statin drugs) is associated with coronary atherosclerosis regression, estimated by intravascular ultrasound (IVUS); (2) to assess the association between dual lipid-lowering-induced changes in low density lipoprotein cholesterol (LDL-C) and non-high-density-lipoprotein cholesterol (non-HDL-C) levels and atherosclerosis regression.

**Methods:**

A meta-analysis including trials of non-statin lipid-lowering therapy, reporting LDL-C, non-HDL-C and total atheroma volume (TAV) with a minimum of 6 months of follow-up was performed. The primary endpoint was defined as the change in TAV measured from baseline to follow-up, comparing groups of subjects on statins alone versus combination of statin and non-statin drugs. The random-effects model and meta-regression were performed.

**Results:**

Eight eligible trials of non-statin lipid-lowering drugs (1759 patients) were included. Overall, the dual lipid-lowering therapy was associated with a significant reduction in TAV [− 4.0 mm^3^ (CI 95% -5.4 to − 2.6)]; I^2^ = 0%]. The findings were similar in the stratified analysis according to the lipid-lowering drug class (ezetimibe or PCSK9 inhibitors). In the meta-regression, a 10% decrease in LDL-C or non-HDL-C levels, was associated, respectively, with 1.0 mm^3^ and 1.1 mm^3^ regressions in TAV.

**Conclusion:**

These data suggests the addition of ezetimibe or PCSK9 inhibitors to statin therapy results in a significant regression of TAV.

Reduction of coronary atherosclerosis observed with non-statin lipid-lowering therapy is associated to the degree of LDL-C and non-HDL-C lowering. Therefore, it seems reasonable to achieve lipid goals according to cardiovascular risk and regardless of the lipid-lowering strategy used (statin monotherapy or dual treatment).

## Introduction

Coronary plaque regression has a significant positive correlation with low density lipoprotein cholesterol (LDL-C) and non-high-density-lipoprotein cholesterol (non-HDL-C) reduction [1]. Multiple diagnostic methods have been used to assess the composition of atherosclerotic plaque. Coronary intravascular ultrasound (IVUS) has been used in most of the studies that evaluate the impact of pharmacological interventions on the progression/regression of atherosclerosis. On the other hand, the regression of atherosclerosis measure by IVUS has been a subrogated endpoint of clinical cardiovascular events [2, 3].

The use of statins has been and continues today to be the cornerstone of risk management of cardiovascular disease. The robust evidence showing a reduction of cardiovascular events has made statins essential in a variety of clinical conditions with elevated cardiovascular risk [4–6]. In the same way, several studies have demonstrated that statin therapy promotes coronary atheroma stabilization and regression in patients with acute coronary events or stable coronary disease [7–11].

Despite reductions in LDL-C with statins, many people still experience cardiovascular events (cardiovascular residual risk). Dual lipid-lowering therapy with ezetimibe or inhibitors of proprotein convertase subtilisin kexin type 9 (PCSK9) has been shown to be more effective than statin monotherapy in high-risk patients with coronary artery disease [12, 13]. In recent years, many studies have investigated the reduction of LDL-C with non-statin drugs and Its impact on atherosclerosis regression. However, the studies were mostly small and their results were not always robust [14–21]. A previously published meta-analysis showed an association between the regression of atheroma volume with the addition of ezetimibe [22]. Nevertheless, this study did not include a meta-regression to assess the relationship between the decrease in lipid markers achieved with dual lipid-lowering therapy and plaque regression. Moreover, it didn’t evaluate non-statin drugs such as PCSK9 inhibitors. The dual therapy would be successful if it reduces an adequate percentage in lipid levels and, consequently, attains regression in the volume of atherosclerotic plaque. The hypothesis of this investigation was that an additional decrease in cholesterol levels with a non-statin therapy would result in atherosclerosis regression, being greater with more intensive decrease in lipid levels. Therefore, the objectives of the present meta-analysis were: (1) to define if a more aggressive dual lipid-lowering therapy with non-statin drugs is associated with a regression in coronary atherosclerosis estimated by IVUS; (2) to assess the association between dual lipid-lowering-induced changes in LDL-C and non-HDL-C levels and regression of atherosclerosis.

## Material and methods

### Data extraction and quality assessment

This meta-analysis was performed according to the Preferred Reporting Items for Systematic Reviews and Meta-Analyses (PRISMA) guidelines for reporting systematic reviews [23]. A literature search was performed that identified clinical trials of non-statin drugs that are recommended by the current cholesterol guidelines based on the results of clinical trials that showed efficacy in the reduction of cardiovascular events, and published between January 1990 and January 2020 in English. Two independent reviewers searched the electronic PubMed/MEDLINE, Embase and Cochrane Controlled Trials databases using the following terms: “endovascular ultrasound”, “IVUS”, “atherosclerosis regression”, “lipid-lowering therapy”, “ezetimibe” and “PCSK9 inhibitors”.

All the analyzed studies meet the following inclusion criteria: a) Comparisons of efficacy for a statin monotherapy versus statin plus non-statin therapy (ezetimibe or PCSK9 inhibitor therapy); b) Follow-up duration ≥6 months; c) IVUS used as modality for measuring changes in atheroma volume; c) Reporting of change in atheroma between baseline and follow-up, expressed as mean delta total atheroma volume (TAV); d) Reporting the change in lipids values between baseline and follow-up.

The primary endpoint of the study was defined as the change in TAV measured from baseline to follow-up, comparing groups of subjects on statins alone versus combination of statin and non-statin drugs.

The dual therapy was used to investigate whether the additional decrease in LDL-C or non-HDL-C was associated with a regression in the volume of atheroma in the evaluated studies. Basal population risk is the main criterion by which treatment without statins should be considered;in that sense, all selected studies included high or very high cardiovascular risk patients. Only 3 studies evaluated in this meta-analysis determined as an inclusion criteria having a baseline level of LDL-C > 100 mg/dl.

When the summary/dispersion measures used to report the difference in TAV between the arms were not mean and standard deviation, conversion tools previously suggested by the literature were used [24].

Potential risks of bias were evaluated, using the Cochrane tool developed for this purpose [25]. This tool assesses bias in different domains: random sequence generation (selection bias); allocation concealment (selection bias); blinding of participants and study staff (performance bias); blinding of outcome assessors (detection bias); incomplete results data (attrition bias); selective reporting of results (reporting bias); and other sources of bias. Each domain was rated as “High”, “Low” or “Unclear” depending on the judgment of each author following the recommendations.

### Meta-analysis and meta-regression analyses

The summary effect of non-statin lipid- lowering drugs on the TAV was estimated. Exploratory meta-regression analyses were performed to examine the potential associations between C-LDL and non-HDL-C reduction and the effect sizes of non-statin lipid-lowering drugs on atheroma regression.

### Statistical analysis

Measures of effect size were expressed as mean difference, and the I^2^ statistic was calculated to quantify between-trial heterogeneity and inconsistency. Because studies differ in their lipid-modifying regimens and effect sizes, a random-effects model was chosen. However, to assess the relationship between differences in LDL-C and non-HDL-C reduction and variations in mean difference of atheroma volume, a mixed-effects meta-regression model was performed. To compare mean effects between subgroups, a Z test was used. The level of statistical significance was set at a two-tailed alpha of 0.05. Statistical analyses were performed using the R software for statistical computing version 3.5.1 with additional specific packages [26].

### Sensitivity analyses

Through this analysis, the results of the meta-analysis were replicated excluding in each step one of the studies included in the review. The analysis was considered robust when the results obtained were similar.

Analysis of publication bias: A funnel plot using the standard error (SE) for mean difference was created, and Begg and Mazumdar rank correlation were also performed. In addition, Egger’s regression intercept tests were done.

## Results

**Fig. 1:**
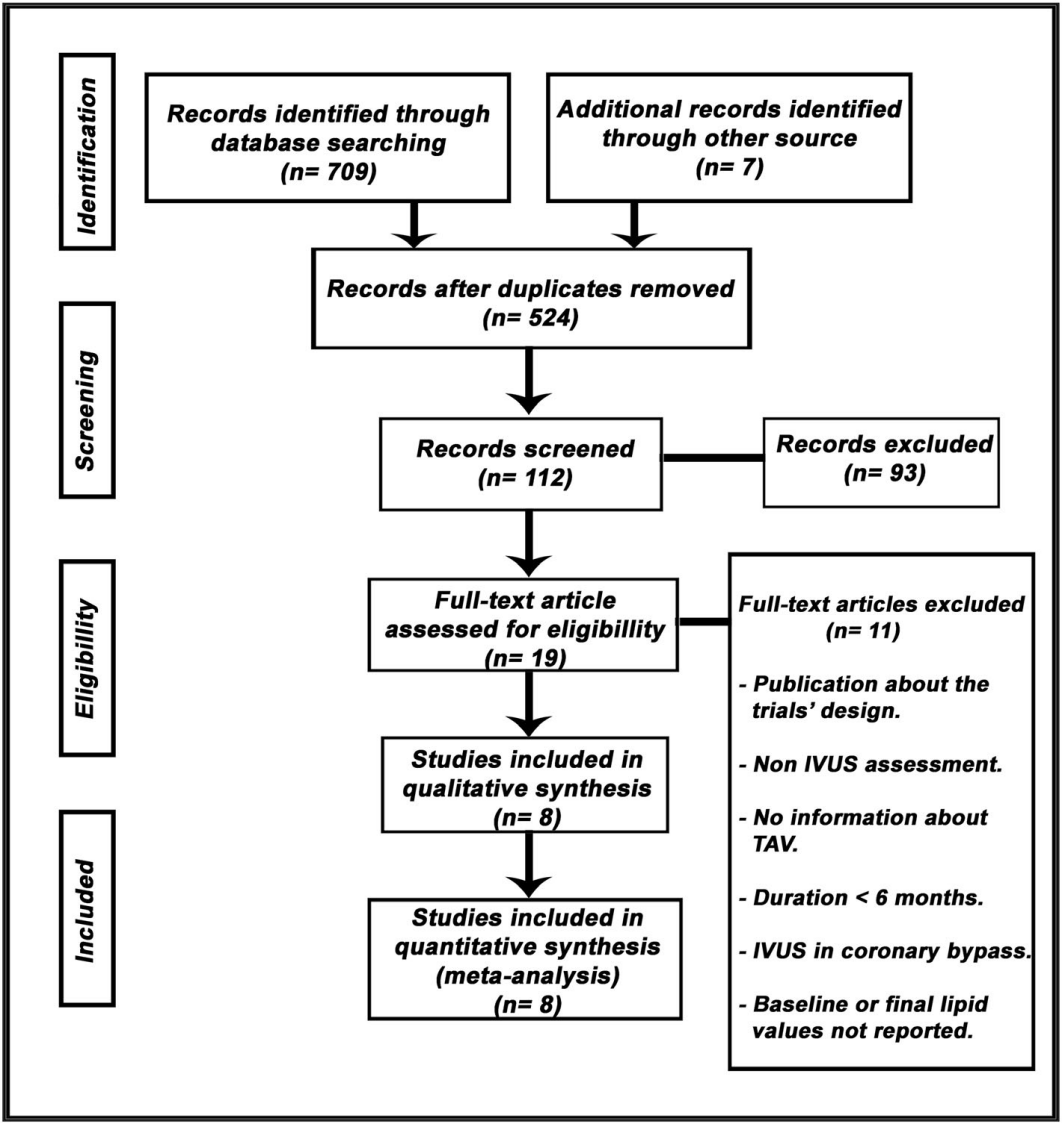
Flow diagram of the study screening process. TAV: total ateroma volumen; IVUS: intravascular ultrasound

Eight eligible trials of non-statin lipid-lowering drugs, including 1759 patients, were identified and considered eligible for the analyses. There was a total of 879 subjects allocated to receive dual lipid-lowering therapy (statin plus ezetimibe or PCSK9 inhibitors) and 880 subjects allocated to the respective control arms (statin monotherapy). A flow diagram of the study’s screening process has been shown in Fig. 1.

**Fig. 2:**
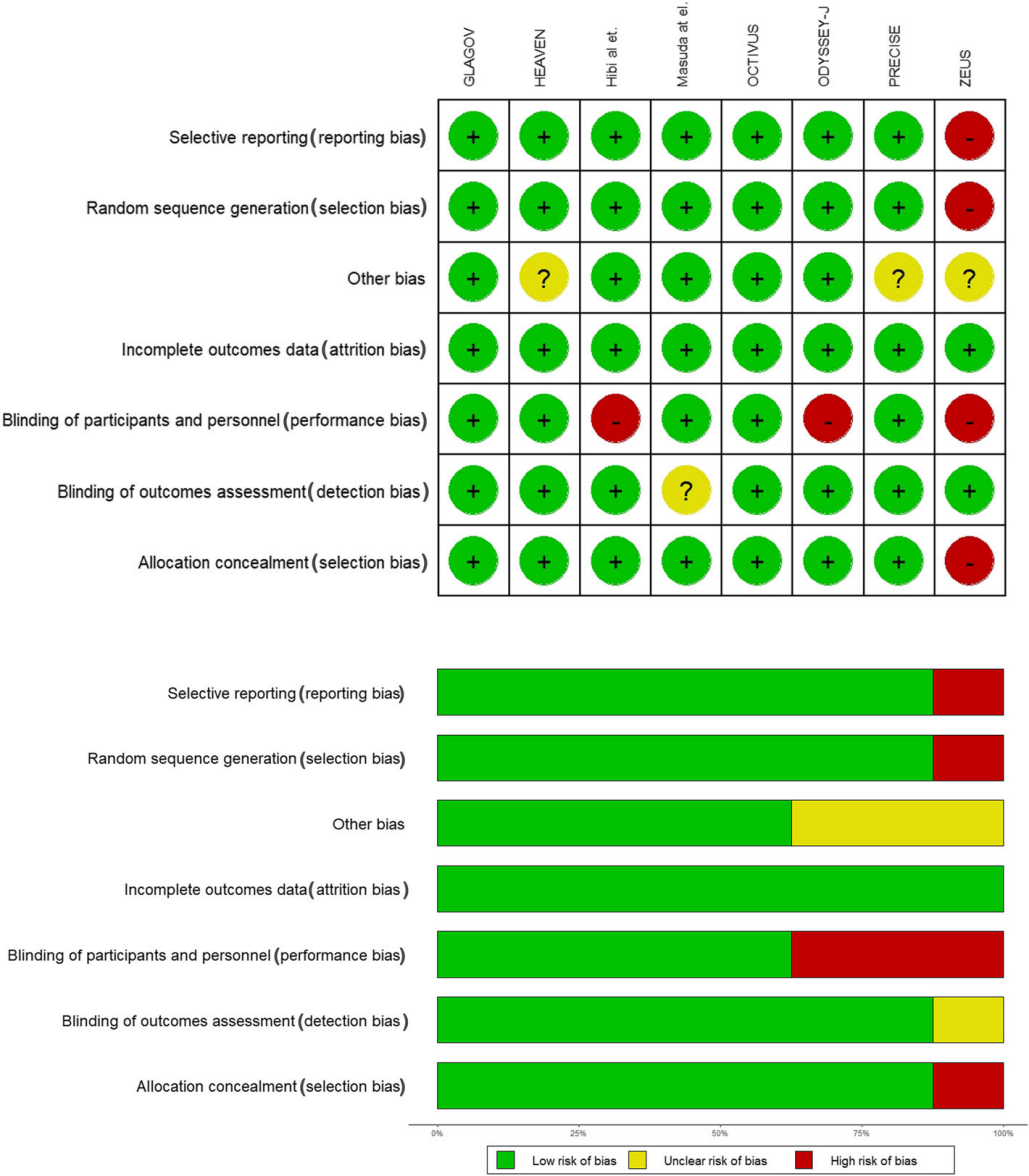
**a** Individual bias assessment of included studies. **b** Summary bias assessment of included studies

Seven studies were randomized and all of the trials evaluated had a control arm. The quality of the studies evaluated can be seen in Fig. 2.

Three studies included patients with chronic coronary heart disease (HEAVEN, Masuda et al., GLAGOV) and other four studies evaluated subjects after an acute coronary syndrome (OCTIVUS, ZEUS, Hibi et al., ODYSSEY-J). The additional study accepted both options as inclusion criteria (PRECISE-IVUS). Six studies that evaluated the addition of ezetimibe and two studies that analyzed the additional effect of PCSK9 inhibitors were included. In studies that included patients with acute coronary syndrome, the IVUS measurement was performed in a different coronary segment than the culprit lesion.

The follow-up ranged between 6 and 19 months. The characteristics of the studies included in the analysis can be seen in Table 1**.**

**Table 1:** Characteristics of the studies included in the analysis

*Study*	*Publication year*	*Population*	*Follow-up (months)*	*Design*	*Dual lipid-lowering therapy*				*Statin monotherapy*			
					***N***	***Treatment***	***Baseline LDL-C***	***Baseline non-HDL-C***	***N***	***Treatment***	***Baseline LDL-C***	***Baseline non-HDL-C***
HEAVEN [14]	2012	SAP	12	Single-blinded. RCT	42	Atorvastatin 80 mg/day + Ezetimibe 10 mg/day	119.9	143.1	47	Standard statin therapy	104.4	131.5
OCTIVUS [15]	2016	ACS	11.6	Double-blinded. RCT	39	Atorvastatin 80 mg/day + Ezetimibe 10 mg/day	142.9	162.1	41	Atorvastatin 80 mg/day + Placebo	158.3	177.6
ZEUS [16]	2014	ACS	6	Open label. Non RCT	50	Atorvastatin 20 mg/day + Ezetimibe 10 mg/day	116.2	137.5	45	Atorvastatin 20 mg/day	114.3	137.8
Hibi et al. [17]	2018	ACS	10	Open label. RCT	50	Pitavastatin 2 mg/day + Ezetimibe 10 mg/day	123.0	146.0	53	Pitavastatin 2 mg/day	126.0	150.0
PRECISE [18]	2015	ACS/SAP Basal LDL-C > 100 mg/dl	10.1	Single-blinded. RCT	100	Atorvastatin^a^ + Ezetimibe 10 mg/day	109.8	136.2	102	Atorvastatin^a^	108.3	132.7
Masuda et al. [19]	2015	SAP Basal LDL-C > 100 mg/dl	6	Open label. RCT	21	Rosuvastatin 5 mg/day + Ezetimibe 10 mg/day	131.8	151.4	19	Rosuvastatin 5 mg/day	123.0	146.2
GLAGOV [20]	2016	SAP	19	Double-blinded. RCT	484	Statin + Evolucumab 420 mg M	92.6	119.4	484	Statin + Placebo	92.4	120.8
ODYSSEY-J [21]	2019	ACS. Basal LDL-C > 100 mg/dl	9	Open label. RCT	93	Statin^b^ + Alirocumab 75/150 mg Q2W	97.9	124.1	89	Statin^b^	95.7	124.4

*ACS* acute coronary syndrome, *M* one monthly, *Q2W* every 2 weeks, *RCT* randomized clinical trial, *SAP* stable angina pectoris

^a^Atorvastatin was increased by titration with the usual dose range with a treatment goal of LDL-C < 70 mg/dl

^b^Standard-of-care

**Fig. 3:**
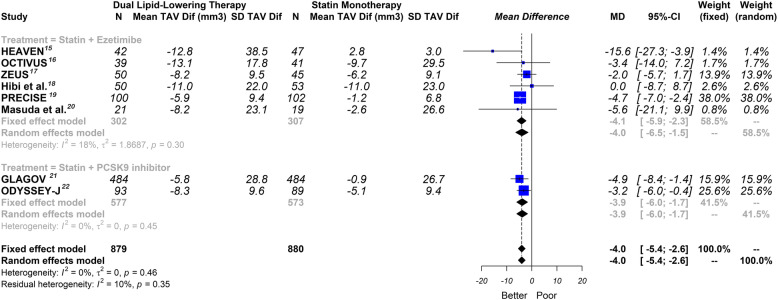
Effect of non-statin lipid-lowering therapy on total atheroma volume. Global and subgroup drug analysis. Random effects, mean difference, 95% confidence intervals (CI) and I^2^ statistics. TAV: total atheroma volume; Dif: difference

Overall, this meta-analysis showed that dual lipid-lowering therapy was associated with a significant reduction in TAV [− 4.0mm^3^ (CI 95% -5.4 to − 2.6)]; *P* < 0.0001; I^2^ = 0%] Fig. 3.

In the stratified analysis according to the lipid-lowering drug class, the findings were similar: (1) ezetimibe group: [− 4.0 mm^3^ (CI 95% -6.5 to − 1.5)]; *P* = 0.0018; I^2^ = 18%]; (2) PCSK9 inhibitor group: [− 3.9 mm^3^ (CI 95% -6.0 to − 1.7)]; *P* < 0.0001; I^2^ = 0%] Fig. 3.

**Fig. 4:**
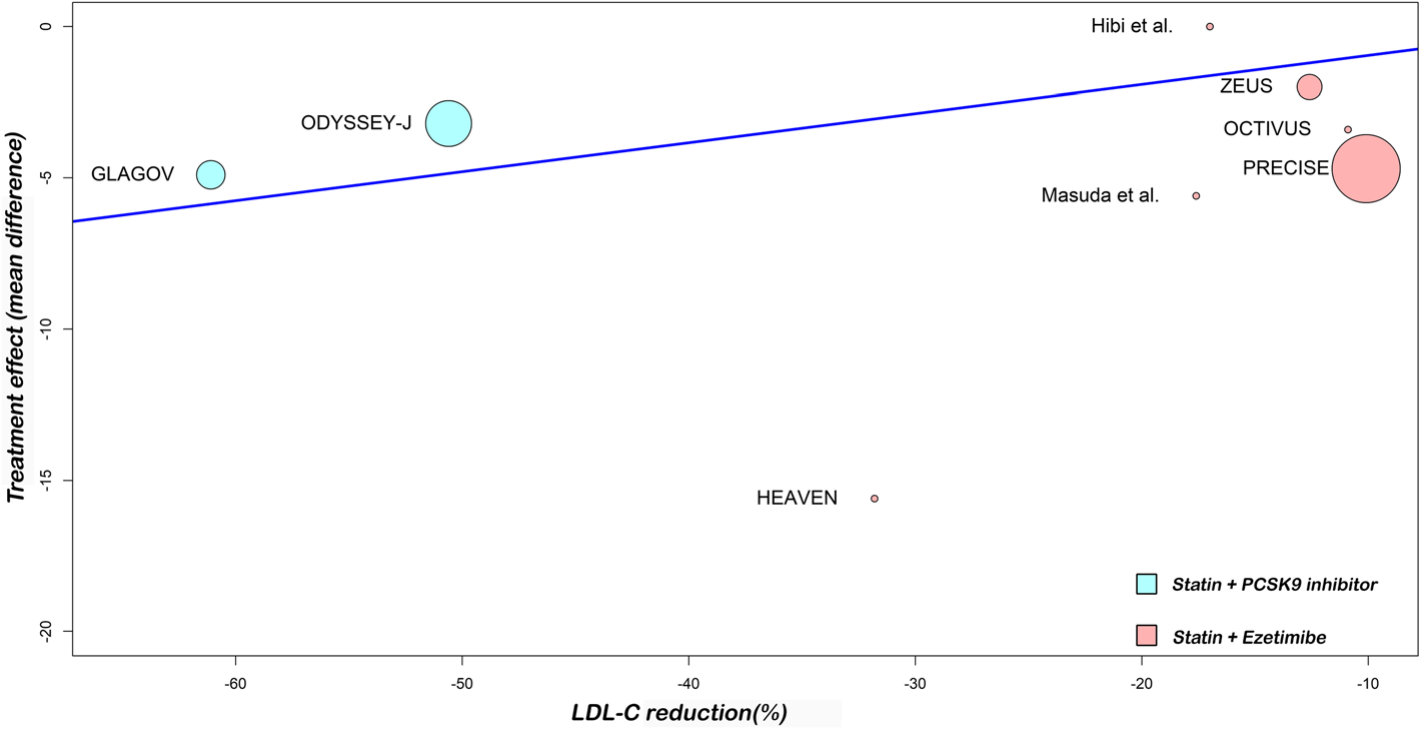
Random-effects meta-regression analyses: Association between the difference in percentage LDL-C reduction among treatment arms and treatment effect (total atheroma volume regression)

**Fig. 5:**
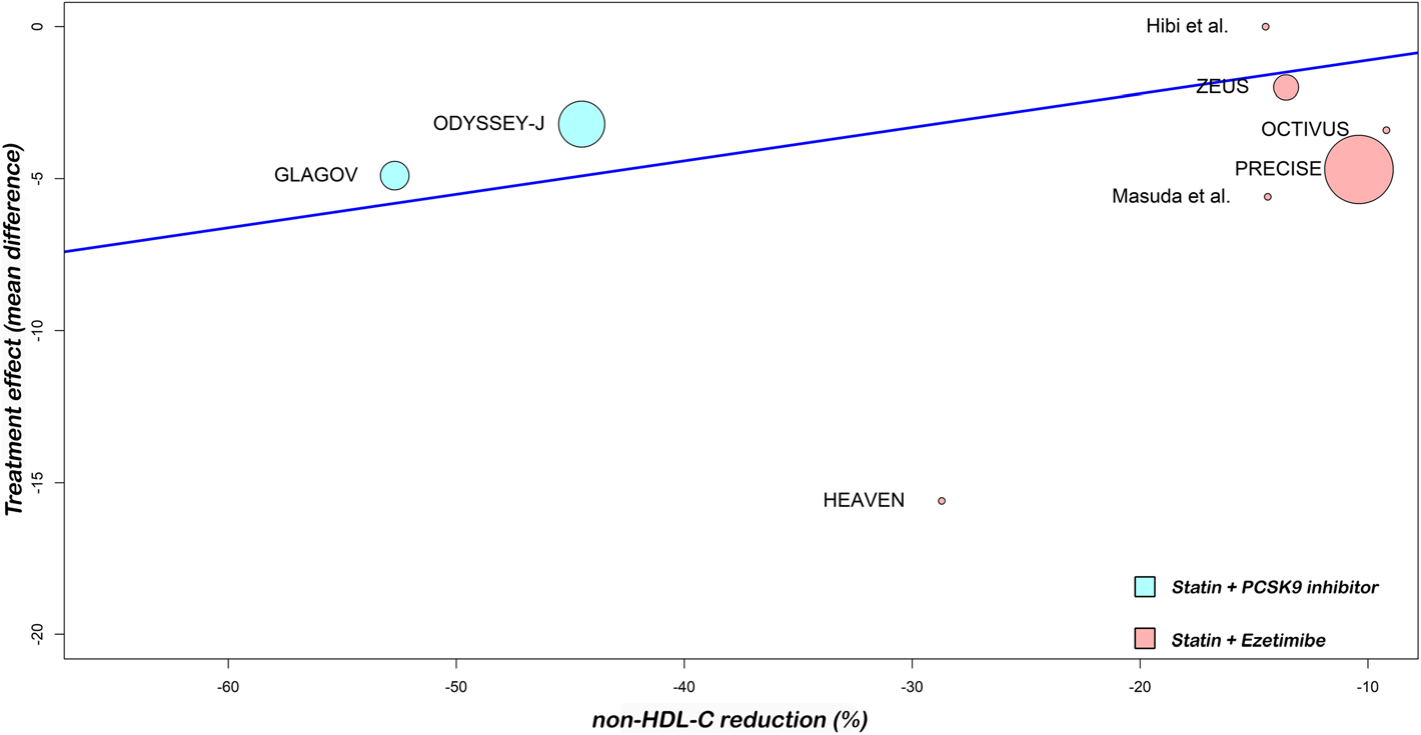
Random-effects meta-regression analyses: Association between the difference in percentage non-HDL-C reduction among treatment arms and treatment effect (total atheroma volume regression)

In the meta-regression, the LDL-C reduction associated with dual lipid-lowering therapy was associated with a significant reduction in TAV (*P* = 0.0083). Furthermore, when we analyzed the non-HDL-C, meta-regression showed similar results (*P* = 0.0057). In addition, a 10% decrease in LDL-C or non-HDL-C levels, was associated, respectively, with 1.0 mm^3^ and 1.1 mm^3^ regressions in TAV. Figures 4 and 5 show meta-regression analysis by lipids values reductions.

**Fig. 6:**
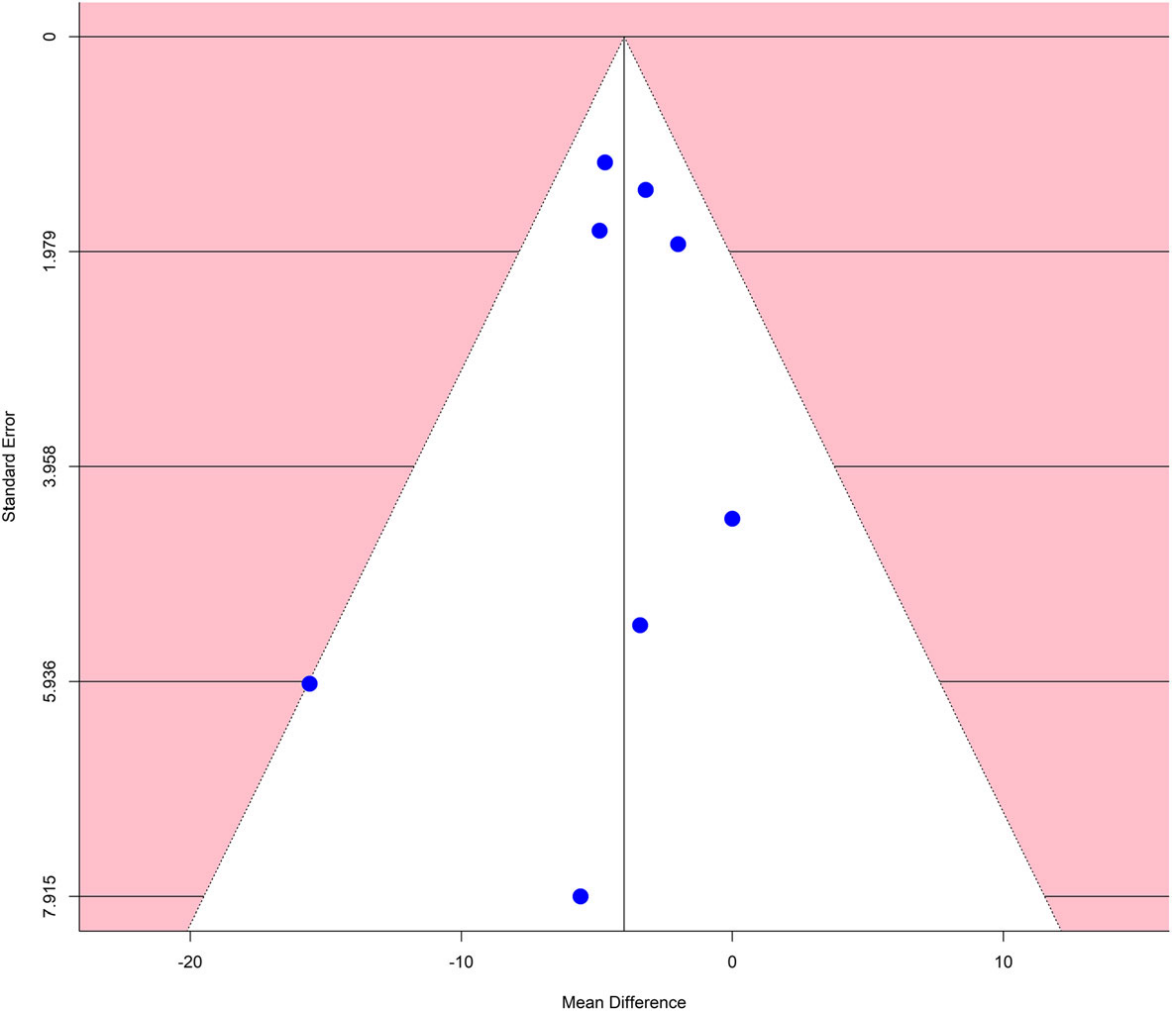
Funnel plot to assess publication bias

The funnel plot of standard error by mean difference of endpoints did not suggest publication bias Figure 6. In the same way, Begg and Mazumdar’s test for rank correlation gave a *P* value of 0.8046, not indicating possible publication bias. In addition, Egger’s regression intercept tests gave a *P* value of 0.6876.

**Fig. 7:**
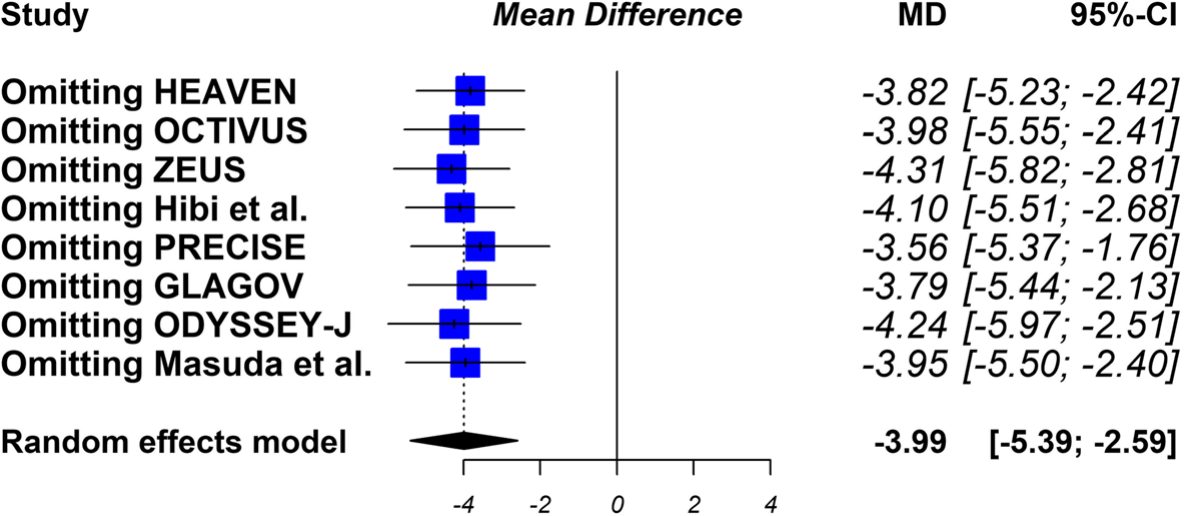
Sensitivity analysis. After replicating the results of the meta-analysis, excluding in each step one of the studies included in the review, the results obtained are similar

The sensitivity analysis showed that the results were robust Fig. 7..

## Discussion

In this meta-analyses, dual lipid-lowering treatment (statin plus ezetimibe or PCSK9 inhibitors) compared with statin monotherapy was associated with greater reduction in TAV. The results were consistent in the global and lipid-lowering drugs subgroups analysis, suggesting that the decrease in LDL-C itself would be more relevant than the pharmacological mechanism that generates it. There is strong evidence of the relationship between LDL-C levels, the regression of atherosclerotic plaque and the reduction of cardiovascular events [1, 5]. Statins play a role in plaque regression with reduction in lipid content. These medications stabilize atherosclerotic plaque with thickened fibrous layers and macrocalcification [8].

Ezetimibe, an inhibitor of the Niemann-Pick C1-like 1 cholesterol transporter, is a relatively new drug for LDL-C-lowering therapy [27]. Combination therapy with a statin and ezetimibe produced better clinical outcomes than statin monotherapy in the IMPROVE-IT study [12]. Similarly, PCSK9 inhibitors are new pharmacologic agents that have an incremental effect on lowering LDL-C in statin-treated patients, combined with an excellent safety profile [28]. In the recent FOURIER and ODYSSEY OUTCOMES trials, PCSK9 inhibition produced a relevant reduction in serum LDL-C levels by suppressing LDL-C receptor degradation and, consequently, has demonstrated clinical efficacy, in addition to statin therapy, in reducing cardiovascular events in patients with clinical evident atherosclerotic disease [13, 29].

The effect of lipid reduction on the atheroma plaque regression was mainly evaluated in statin trials. For example, one of the pioneering investigations, the REVERSAL study, showed regression of the statin-mediated coronary plaque when the decrease in LDL-C level exceeded 50% [30].

The role of ezetimibe in atherosclerosis regression was initially uncertain. The ENHANCE study did not find significant changes in the intima-media thickness in patients with familiar hypercholesterolemia treated by simvastatin with and without ezetimibe [31]. Nevertheless, beyond some methodological limitations of this study, the use of carotid ultrasound to assess the regression of atherosclerosis has been displaced by IVUS. A recent meta-analysis found no significant association between LDL-C reduction and progression of atherosclerosis estimated by carotid intima-media thickness [32].

Atherosclerotic plaque regression and conversion to a stable phenotype is possible with intensive statin therapy and can be demonstrated in patients using a variety of non-invasive and invasive imaging modalities [33]. The use of IVUS in the present analysis to evaluate atheroma volume is a globally established method to evaluate the vascular effect of lipid-lowering therapy. Previously, Mirzaee et al. showed that the addition of ezetimibe to statin therapy is effective in reducing total atheroma volume assessed by IVUS [22]. However, they did not evaluate other non-statin drugs such as PCSK9 inhibitors. Experimental studies have suggested that PCSK9 might directly promote inflammatory processes contributing to atherosclerosis [34]. Likewise, although it is widely accepted that the association between PCSK9 and atherosclerosis is dependent on PCSK9-mediated modulation of LDL metabolism, there is a lot of evidence suggesting that PCSK9 may also exert direct cholesterol-independent pro-atherosclerotic effects [35]. However, a recent meta-analysis failed to demonstrate an effect of PCSK9 inhibitors on high sensitivity C-reactive protein concentrations. This led to questioning the impact of these drugs on systemic inflammation [36]. Two recent trials have evaluated the effect of monoclonal antibodies on atherosclerosis regression. The GLAGOV trial reported the effectiveness of the PCSK9 inhibitor (evolocumab) compared with statin alone, on plaque regression at the LDL-C level of 36 mg/dL, further confirming “the lower the better” theory [20]. The ODYSSEY J-IVUS trials showed that alirocumab treatment over 36 weeks resulted in a numerically greater but not statistically significant percentage reduction in TAV [21]. The lack of a statistically significant difference in the primary efficacy endpoint observed in this study needs to be considered in light of several specific factors of the study design, such as the limited sample size and the short duration of treatment period.

Consequently, it is essential to strengthen the evidence on these new medications. The addition of a PCSK9 inhibitor to a statin regimen has been shown to further reduce LDL-C levels by 43 to 64% [37], and its use could increase considerably since in usual clinical practice, many patients do not reach the goals proposed by combining statins with ezetimibe [38]. Likewise, the latest published guidelines have recommended the use of ezetimibe and PCSK9 inhibitors as additional medications to reduce the residual risk of patients at higher risk [39, 40]. Therefore, we consider the evaluation of the impact of both drugs on the regression of atherosclerosis as a very important fact.

This is the first meta-analysis to demonstrate that the combination of statin and ezetimibe or PCSK9 inhibitors therapy is associated with a significantly greater reduction in TAV, compared with statin monotherapy.

Another point of this study is that it evaluates through a meta-regression analysis the effect of lipid levels reduction generated by dual lipid-lowering therapy on the regression of atherosclerosis.

A large body of evidence supports a central role for LDL-C lowering in the prevention of atherosclerotic cardiovascular disease [41]. It is proven that the process of atherosclerosis strongly depends on LDL-C, but whether or not this dependence is similar in statin regimens and dual therapy regimens is less defined [17, 18]. It is therefore reasonable to think that the effects of the ezetimibe-statin combination therapy can vary according to the patient sample (e.g. statin naïve versus statin pretreated patients; acute coronary syndrome versus chronic coronary disease), and time of assessment of outcome. In a subanalysis of the PRECISE-IVUS trial, the IVUS endpoints were compared according to the presence or absence of statin pretreatment [42]. The atorvastatin/ezetimibe combination showed a significant stronger reduction in atheroma volume, compared with atorvastatin alone, in patients with statin pretreatment. Compensatory increase in cholesterol absorption observed in statin-treated patients might attenuate the inhibitory effects of statins on coronary plaque progression.

The results of this meta-analysis showed that with the lowest LDL-C levels achieved with dual lipid lowering therapy, a greater reduction in plaque volume was observed. These results suggested that the decrease in LDL-C itself would be more relevant than the mechanism that generates it. In that sense, a recent subanalysis of the PRECISE-IVUS study conducted in patients with acute coronary syndrome concluded that the regression of coronary atherosclerotic plaque was associated more with the decrease in LDL-C than with the established lipid-lowering therapy (high-intensity statins or combined therapy) [43].

The called “LDL hypothesis” assumes that reducing LDL-C levels, regardless of the means, should produce a corresponding reduction in cardiovascular events. An alternative theory, referred as “statin hypothesis”, proposes that statins have a unique efficacy in atherosclerotic vascular disease that is not shared by other lipid-modifying agents. Considerable evidence supports the LDL hypothesis, including several epidemiologic studies and clinical trials of both statins and non-statin lipid-modifying agents. The Cholesterol Treatment Trialists’ (CTT) meta-analysis, that included randomized trials of statins, found that, on average, a reduction of 1 mmol per liter in LDL-C levels yields a consistent 23% reduction in the risk of major coronary events over 5 years [6]. Similarly, in the IMPROVE-IT and the FOURIER studies the extent of benefit afforded by the statin-ezetimibe or statin-evolocumab combination respectively, was consistent with that seen in the CTT meta-analysis, with a similar reduction in cardiovascular events according to the degree of LDL-C lowering [12, 29].

The non-HDL-C comprises cholesterol carried by all potentially atherogenic particles, is simpler, more convenient and more predictive than LDL-C [44]. The present study also evaluated the impact of this lipid marker on atheroma regression, showing similar findings to the C-LDL analysis.

The process of plaque regression by aggressive LDL-C lowering therapy could also stabilize the unstable plaque and reverse the positive remodeling of the vessel wall [45]. As combination therapy with a statin and either ezetimibe or PCSK9 inhibitors lowers LDL-C levels beyond that achieved with statin monotherapy, dual lipid-lowering treatment strategy may have additional protective cardiovascular effects [46]. The findings of this meta-analysis align with these concepts. At present, dual therapy is recommended to be administered in patients with high or very high cardiovascular risk who, despite maximum tolerated dose of statins, do not reach the recommended lipid targets. Both ezetimibe and PCSK9 inhibitors are usually well tolerated with few adverse effects. Continued therapy should be sustained in such cases.

## Limitations

This meta-analysis presents several limitations. First, they are related with clinical heterogeneity (popular characteristics, different schemes of lipid-lowering therapy, different follow-up)and the number of patients included in most of the studies was low. In fact, the pathophysiological mechanisms involved in acute coronary syndrome are not exactly the same as in chronic coronary heart disease. However, the statistical heterogeneity was low and the results were robust when performing the sensitivity analysis, including when the sensitivity analysis was done only with randomized clinical trials. Second, the analysis included only trial-level data without having the individual data. Third, the meta-regression analysis was performed with 8 studies, when some authors suggest doing it with 10 or more. We consider that the number of studies evaluated approached the suggested number, being also the total of the available evidence at present. Likewise, meta-regression in our study is a complement to the main result that is meta-analysis. Fourth, this study was not designed to assess the cost effectiveness of non-statin therapy. New studies in this area should be developed to answer this question. Finally, we did not perform the analysis with another primary endpoint, such as the percentage of atheroma volume (PAV), because these data was not informed in all the original publications.

## Conclusion

This data suggest the addition of ezetimibe or PCSK9 inhibitors to statin therapy results in significantly increased regression of TAV. When the LDL-C and non-HDL-C levels reached were lower, the observed effect on atherosclerosis regression was also greater. The process of plaque regression by aggressive LDL-C and non-HDL-C lowering therapy with non-statin drugs can occur. The decrease in LDL-C and non-HDL-C by themselves would be more relevant than the mechanism that generate it and would explain why using these drugs has an additional protective cardiovascular effect. Future lipid lowering drugs should also demonstrate their impact on the regression of atherosclerosis.

## Data Availability

The data in the current paper are publicly available since this a meta-analysis conducted on the basis of the cited literature.

## Abbreviations

TAV: Atheroma volume
PCSK9: Proprotein convertase subtilisin kexin type 9
IVUS: Intravascular ultrasound
LDL-C: Low density lipoprotein cholesterol
non-HDL-C: Non-high-density-lipoprotein cholesterol
PAV: Percentage of atheroma volume
